# SARS-CoV-2 Direct Detection Without RNA Isolation With Loop-Mediated Isothermal Amplification (LAMP) and CRISPR-Cas12

**DOI:** 10.3389/fmed.2021.627679

**Published:** 2021-02-17

**Authors:** Alfredo Garcia-Venzor, Bertha Rueda-Zarazua, Eduardo Marquez-Garcia, Vilma Maldonado, Angelica Moncada-Morales, Hiram Olivera, Irma Lopez, Joaquin Zuñiga, Jorge Melendez-Zajgla

**Affiliations:** ^1^Cancer Functional Genomics Laboratory, Instituto Nacional de Medicina Genómica, Mexico City, Mexico; ^2^Molecular Biology Unit, Instituto Nacional de Enfermedades Respiratorias, Mexico City, Mexico; ^3^Epigenetics Laboratory, Instituto Nacional de Medicina Genómica, Mexico City, Mexico; ^4^Instituto Nacional de Referencia Epidemiológica, Mexico City, Mexico

**Keywords:** COVID-19, SARS-CoV-2, diagnosis, LAMP, CRISPR-Cas12a

## Abstract

As to date, more than 49 million confirmed cases of Coronavirus Disease 19 (COVID-19) have been reported worldwide. Current diagnostic protocols use qRT-PCR for viral RNA detection, which is expensive and requires sophisticated equipment, trained personnel and previous RNA extraction. For this reason, we need a faster, direct and more versatile detection method for better epidemiological management of the COVID-19 outbreak. In this work, we propose a direct method without RNA extraction, based on the Loop-mediated isothermal amplification (LAMP) and Clustered Regularly Interspaced Short Palindromic Repeats-CRISPR associated protein (CRISPR-Cas12) technique that allows the fast detection of SARS-CoV-2 from patient samples with high sensitivity and specificity. We obtained a limit of detection of 16 copies/μL with high specificity and at an affordable cost. The diagnostic test readout can be done with a real-time PCR thermocycler or with the naked eye in a blue-light transilluminator. Our method has been evaluated on a small set of clinical samples with promising results.

## Introduction

The COVID-19 global outbreak caused by the virus SARS-Cov-2 is one of the major global concerns due to its rapid spread around the world. The official numbers on November 07, 2020, are 49,636,193 confirmed cases and 1,247,187 deaths on the entire planet (Johns Hopkins University, https://coronavirus.jhu.edu/map.html; last access on November 07).

The precise and rapid screening of possible infected subjects is one of the major strategies to contain the COVID-19 outbreak, to reduce the impact on the mortality of the population and avoid hospital saturation. However, the current diagnostic strategies mainly consist of qRT-PCR analysis, which has drawbacks as it requires RNA isolation, and thus takes several hours to obtain a result. These aspects have hindered the applicability of qRT-PCR systems to efficiently contain the COVID-19 outbreak (1).

For these reasons, it is of capital importance to develop novel diagnostic techniques, that improve cost, increase the number of the diagnostic facilities able to perform assays, and the time needed to obtain a result. Loop-mediated Isothermal Amplification (LAMP) technology allows the specific amplification of a target DNA or RNA sequences at a single temperature (in contrast with the three steps required by PCR), allowing a continuous target amplification, making it more versatile than PCR. LAMP has been previously used in the diagnosis of different pathogens at low cost and with great sensitivity and specificity (2–5).

Recently, Zhang et al. (6) used LAMP technology for the specific detection of SARS-CoV-2, observing promising results that could have a direct application in the current COVID-19 outbreak. These results led us to take advantage of the versatility and sensitivity of LAMP to standardize a diagnostic kit for SARS-CoV-2 detection for limited-resource settings use.

In this work, we developed a practical SARS-CoV-2 diagnostic method using a LAMP reaction to amplify a specific region of SARS-CoV-2 N gene mRNA and tested three main detection methods: fluorescence accumulation, colorimetric detection, and LbCas12a-mediated detection. The results obtained showed that the best limit of detection (LOD) was obtained using a qPCR thermocycler to measure the fluorescence accumulation over time. Interestingly, the colorimetric approach gave comparable LOD than fluorescence mediated detection. However, its dependence on pH changes makes it unreliable to use with poor buffered samples such as saliva.

## Materials and Methods

### Primers and Probes

We used two different primer sets for our diagnostic assay, one targeting SARS-CoV-2 N gene transcript, and another targeting human RNAseP POP7 mRNA. Primer sequencing was obtained from previous work, and the sequences used are listed in Supplementary Table 1 (7). For controls, we synthesized a fragment of N gene mRNA and RNAseP POP7 mRNA as gene Blocks (gBlocks) (IDT, Cal., USA) (Supplementary Table 2). Additionally, to work with an RNA SARS-CoV-2 N gene control, the gBlock was *in vitro* transcribed using RiboMAX Large Scale RNA Production System kit and following manufacturer instructions (P1280, Promega, MA, USA). The N-gene RNA copy number was determined using its molar concentration and it was serially diluted to obtain a group of known copy number dilutions used for Limited of calculation (LOD) calculation.

For *Lachnospiraceae bacterium* Cas12a (LbCas12) mediated detection, we used two previously designed guide RNAs (gRNAs), targeting the SARS-CoV-2 N gene, and RNAseP POP7 amplicons. The gRNAs sequence is shown in Supplementary Table 3. Also, for LbCas12 fluorescence readout, we used a Taqman probe (5′FAM/3′BHQ1), which is intended to be degraded by the specific activation of LbCas12a.

### LAMP Reactions

Fluorescent LAMP reactions were done using the WarmStart LAMP kit (E1700L, New England Biolabs, NE, USA), and Colorimetric WarmStart LAMP 2X Master Mix (M1800L, New England Biolabs, NE, USA) was used for colorimetric LAMP. All LAMP reactions were done following manufacturers' instructions, per 29 μL reaction we use 2.5 μL of a LAMP primer mix (F3, B3: 2 μM, LF, LB: 4 μM and FIP, BIP: 16 μM), 5 μL of sample or control and MgSO_4_ concentration was increased to 6 mM by adding 1.13 μL per reaction, of a 100 mM solution. For colorimetric and fluorescent LAMP reactions, the amplification was carried by incubating at 65°C for 40 min. For real-time fluorescent detection of LAMP reactions, we used a QuantStudio 3 Real-Time PCR system (ThermoFisher Scientific, MA, USA) thermocycler, using a PCR program of 160 cycles of 15 s each. End-point fluorescence quantification was carried out in a Beckman Coulter DTX 880 multimode detector (Beckman Coulter, Nyon, Switzerland), using the FAM emission and excitation filters. Direct visualization of fluorescence was done in a tabletop transilluminator.

### LbCas12a Detection

For LbCas12a mediated detection we used commercial LbaCas12a (EnGen LbaCas12a, M0653T, NEB, NE) and RNA was synthesized as RNA oligonucleotides by IDT (CA, USA). First, LbaCas12a-gRNAs complexes were generated by incubating LbaCas12a (50 nM, final concentration) and, gRNAs (62.5 nM), in 1X NEBuffer 2.1 at a final volume of 20 μL per reaction. The LbaCas12a-gRNA complexes were incubated 30 min at 37°C, after incubation, the Taqman probe was added at 500 nM final concentration and transferred to ice. After the LAMP reaction is done, 2 μL of each LAMP reaction was added to 20 μL of the LbaCas12a-gRNA complexes, and the reactions were incubated at 37°C for 10 min. The real-time fluorescence acquisition was done using the QuantStudio 3 Real-Time PCR system (ThermoFisher Scientific, MA, USA) thermocycler, using a qPCR program of 80 cycles of 15 s at 65°C. The visual inspection of fluorescence was done using a blue-light transilluminator (Invitrogen, CA, USA).

### *In silico* Alignment

We used blastn from NCBI for the alignment *in silico*, we compare every primer sequence to the main virus and bacteria that causes symptoms similar to covid-19 or are related to the virus SARS-CoV-2 (Candida albicans taxid: 5476, Neisseria meningitidis taxid: 487, pseudomonas aeruginosa taxid: 287, Staphylococcus aureus taxid: 1280, Influenza A taxid: 11320, Influenza B taxid: 11520, bat MERS-like coronavirus taxid: 2664423, MERS-CoV taxid: 1335626, HCoV-SARS taxid: 694009, HCoV-229E taxid: 11137, HCoV-OC43 taxid: 31631, HCoV-NL63 taxid: 277944, and HCoV-HKU1 taxid: 290028).

### ROC Curves and AUC Values

The receiver operating characteristic (ROC) curves and area under the ROC curve (AUC) values were calculated using pROC R package (1.16.2) with the values of Cycle threshold (Ct) of the LOD curves or patient samples evaluation from fluorescent LAMP reactions or the qualitative values of the use of Cas12a variation. The non-amplified cases were considered as Ct = 160. An AUC of 50% is related to a positive result by chance, while we consider a percentage above 90% as acceptable and an accurate result.

### Sample Treatment and RNA Extraction

For the LOD determination, we added a known amount of N gene RNA copies to 8 μL of a healthy donor nasal swab, subsequently adding 2 uL of 5X lysis solution (0.5 M DTT and 5 mM EDTA). The samples were incubated at 42°C for 20 min, followed by 64°C for 5 min. These steps allowed nuclease inactivation and lysis of biological material releasing nucleic acid without extraction. Five microliter of these inactivated samples were added to 20 μL of LAMP reaction and processed as specified before. Also, this simple inactivation method was used for virus inactivation and lysis of samples from patients.

### Patients

The Institutional Review Board (IRB) of the National Institute of Respiratory Diseases (INER) reviewed and approved the protocols for COVID-19 studies. This project was approved by the IRB under the registration number B09-20. All subjects or their legal responsible, particularly in case of critically ill patients, provided written informed consent for these studies, and they authorized the storage of their nasopharyngeal samples at INER repositories for this and future studies. In this study, we did not collect samples from minors/children, only young adults older than 17 years were included.

A total of 34 patient samples were collected at the Molecular Biology “Unidad de Biología Molecular”, from the National Institute for Respiratory Diseases in Mexico City. We collected 31 nasal swabs and 3 saliva samples from individuals that attended to the emergency room of the institute between March 01 and May 31, 2020; coinciding with the rise of the COVID-19 outbreak in Mexico City. RNA extraction was done by the addition of 2 μL of Lysis solution (DTT 0.5 M and EDTA 5 mM) to 8 μL of the sample, followed by incubation at 42°C for 20 min, and 64°C for 5 min. For LAMP reactions, we used 5 μL of the extracted samples for SARS-CoV-2 N gene amplification and 5 μL for POP7 amplification. Real-time fluorescence measurement was done using a StepOnePlus Real-Time PCR system (ThermoFisher, MA, USA) for RT-qPCR and LAMP reactions. The presence of viral transcript was assessed first by a one-step RT-PCR with a modified Berlin method internally standardized, using specific oligonucleotide sequences for the E and RdRp genes (2019-nCoV) and confirmed with the LAMP test.

### Assay for SARS-CoV-2 Detection by a LAMP-LbCas12a Method

The LAMP assay tube contained 24 μL of the mix with the LAMP primers and Bst enzyme. To this tube 5 μL of sample or control was added and incubated for 40 min at 65°C. After this time 2 μL of this reaction was added into the LbCas12a tube containing the enzyme-gRNA complex and the reporter. The reaction was incubated for 10 min at 37°C to observe or measure the fluorescence (Figure 5).

## Results

### Sensitivity and Specificity of SARS-CoV-2 LAMP Detection

To detect the SARS-CoV-2 virus we used two previously published primer sets, one targeting a specific region of SARS-CoV-2 N-gene and a human control set targeting the RNAseP POP7 gene (3). The N-gene set detected the presence of the COVID-19 virus, whereas the POP7 set was a control for the presence of human transcripts. Alignment analysis was performed to evaluate the specificity of the LAMP amplification primers for N-gene. We considered the main pathogens causing respiratory symptoms like those of Covid-19. No cross alignment was found with any virus or bacteria, suggesting high specificity of the sequences used for detection. For amplification detection, we used two main systems: fluorescence, and colorimetry. For the fluorescent detection system, we added an ATTO Intercalating dye with similar absorption/emission spectra than SYBR-green to the LAMP reactions. Fluorescence increase was recorded using a real-time PCR thermocycler, and the final fluorescence with a multi-well plate fluorometer. The colorimetric assay is based on the acidification of the reaction buffer by the amplification system, which can be detected by a colorimetric pH sensor. This detection system has the advantage of no require any additional detection device than the human eye. Also, to improve the applicability of the method to the actual needs of physicians, we standardized a rapid method for sample inactivation and lysis, based on high temperatures and reducing conditions. To this end, we prepared a lysis solution at 5X concentration, added 1 μL of lysis solution per every 4 μL of a nasal swab, and heated the samples at 42°C for 20 min followed by 64°C for 5 min.

**Figure 1:**
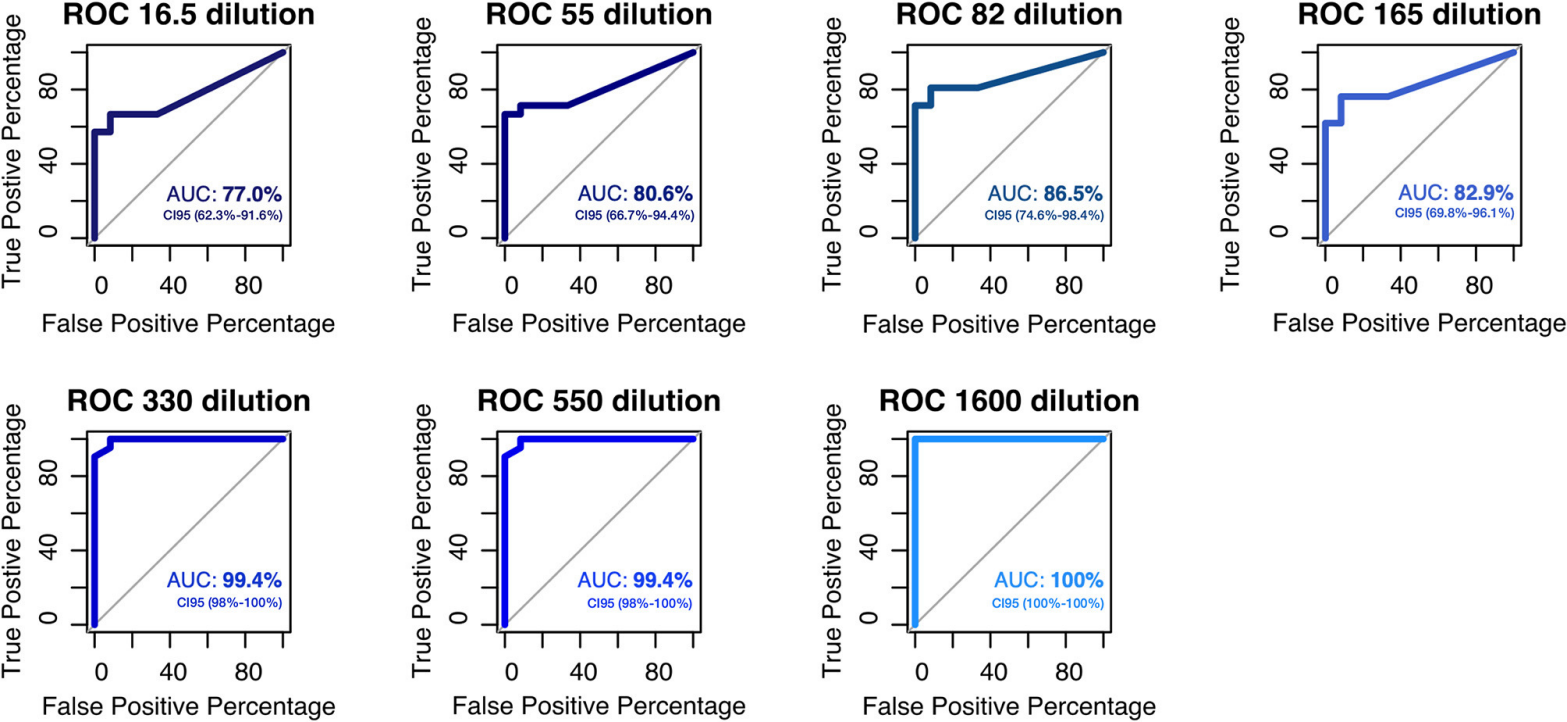
ROC curves and AUC values for each copy number concentration spiked with SARS-CoV-2. The dataset used for the construction of ROC curves and AUC values corresponds to the LOD curves for fluorescent LAMP reactions analyzed using a Real-time thermocycler. From the dilution with 330 copies, we can observe an acceptable accuracy of the test (AUC > 90.0%).

First, we wanted to determine the limit of detection of these assays, for this end, we used a synthetic SARS-CoV-2 N-gene RNA diluted to the specific copy number concentration using a 5 μL of sample volume per reaction (Table 1). Real-time detection of LAMP amplification in a qPCR thermocycler showed a 100% rate of detection (LOD) in samples with 66 copies/μL after 30 min of amplification (Table 2). To enhance the LOD, we increased the reaction time to 50 min. Even when we found a decreased LOD of 6.6 copies/μl, this modification also increased the probability of a false-positive detection, since the proportion of amplificated NTC increased to 40% (Table 2). For this reason, we expanded the number of samples, confirming the LOD with 66 copies/μL after 40 min of amplification (Table 3). The ROC curve analysis showed that the LOD of 66 copies/μL presents a good proficiency in terms of specificity and sensitivity (AUC of 99.4%, CI 95 = 98–100%) (Figure 1).

**Table 1:** The copy number concentration of samples.

**Sample**	**cp/5 μL sample**	**cp/μL**
NTC	0	0
S16.5	16.5	3.3
S33	33	6.6
S55	55	11
S82	85	17
S165	165	33
S330	330	66
S550	550	110
S1000	1,000	200
S1600	1,600	320

*NTC, Non-template control; Cp, copies*.

**Table 2:** Limit of detection of LAMP reaction by real-time fluorescence with 30 and 50 min.

	**Signal Positivity**						
**Samples**	**N1**	**N2**	**N3**	**N4**	**N5**	**Det. at 30 min**	**Det. at 50 min**
NTC	–	–	–	–	–	0%	40%
S16.5	–	–	+	+	+	60%	80%
S33	+	+	+	–	+	80%	100%
S55	+	+	+	+	+	100%	100%
S82	+	+	+	+	+	100%	100%
S165	+	+	–	+	+	80%	100%
S330	+	+	+	+	+	100%	100%
S550	+	+	+	+	+	100%	100%
S1000	+	+	+	+	+	100%	100%
S1600	+	+	+	+	+	100%	100%

*+ is a positive signal*.

*– is a negative signal*.

**Table 3:** Limit of detection of LAMP reaction by real-time fluorescence.

**Signal of positivity (Ct values)**																						
**Sample**	**N1**	**N2**	**N3**	**N4**	**N5**	**N6**	**N7**	**N8**	**N9**	**N10**	**N11**	**N12**	**N13**	**N14**	**N15**	**N16**	**N17**	**N18**	**N19**	**N20**	**N21**	**Det 40 min**
NTC	NS	NS	NS	NS	–	NS	152	158	–	–	–	130	NS	NS	NS	157	–	–	–	–	–	–
S16.5	NS	NS	89	79	86	120	146	123	114	110	NS	102	124	NS	NS	NS	119	129	133	NS	126	67%
S55	78	84	109	96	77	140	123	102	113	NS	114	114	NS	NS	113	NS	129	115	NS	119	NS	71%
S82	95	109	99	106	91	114	99	112	160	120	138	NS	108	NS	142	100	127	NS	104	114	111	86%
S165	89	104	NS	87	115	NS	138	142	134	122	119	106	NS	114	108	107	121	110	NS	NS	129	81%
S330	101	111	118	106	96	130	121	131	104	115	98	95	94	100	95	100	95	106	68	104	104	100%
S550	103	95	85	106	114	130	142	90	118	120	110	106	105	97	107	101	105	85	124	105	77	100%
S1600	25	26	25	26	25	38	38	38	38	39	42	32	32	33	33	32	33	32	33	31	32	100%

*NS, Non signal*.

**Figure 2:**
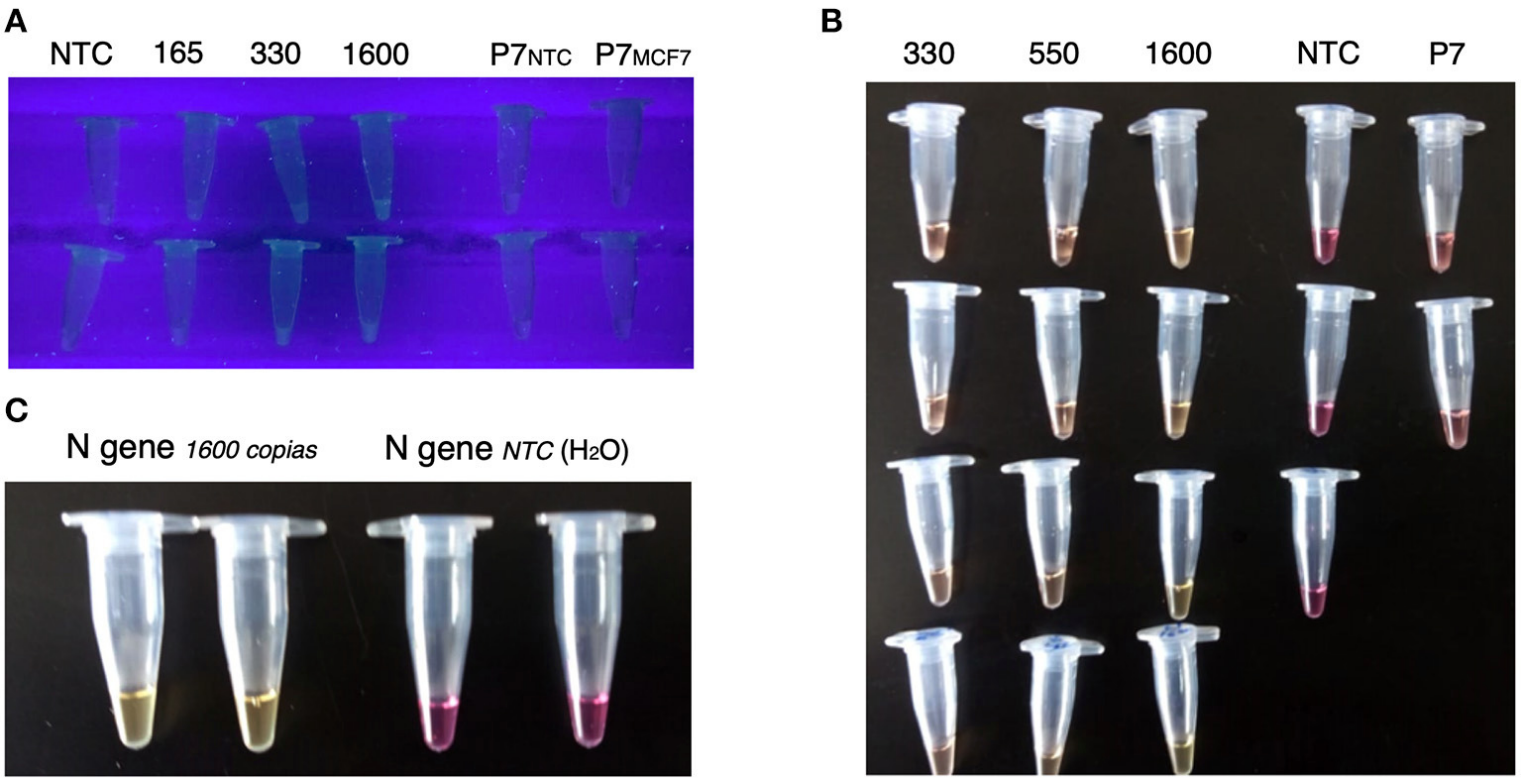
Visual results of fluorescent and colorimetric LAMP systems. **(A)** Representative images of the visual inspection of LAMP fluorescent reactions using a UV transilluminator, fluorescence is perceived in the 66 and 320 copies/5 uL reactions. **(B)** Representative images of the LOD curves of colorimetric LAMP reactions, positive reactions change toward yellow, but negative ones remain pink. **(C)** Representative images of positive and negative colorimetric LAMP results.

The specificity of the N-gene LAMP reaction was assessed using human total RNA samples (MCF-7) without N-gene transcripts (Figure 2A). We observed that MCF-7 samples behave as NTC samples (data not shown). As expected, we observed a specific amplification in MCF-7 samples using the POP7 primer set. Besides, we used a panel of controls, including influenza A/H1N1/pdm09, Influenza B, and other common human coronaviruses like HCoV-NL63, HCoV-HKU1, HCoV-OC43, and HCoV-229E to verify the specificity of the test. All cases were negative to SARS-CoV-2, so there is no cross-reactivity with these viruses.

Next, we analyzed the final fluorescence of LAMP reactions using a multi-well plate fluorometer, using the fluorescein isothiocyanate (FITC) filter. The results showed that final fluorescence detection was less sensitive than real-time fluorescence detection since the LOD obtained for final fluorescence detection was of 1,000 overall copies (Table 4). These results highlight that final fluorescence detection by fluorometer showed a strong downfall in sensitivity and reproducibility.

**Table 4:** Limit of detection of LAMP reaction by final fluorescence.

**Signal positivity**					
**Samples**	**N1**	**N2**	**N3**	**N4**	**% detection**
NTC	–	–	–	–	0%
S16.5	–	–	–	–	0%
S33	+	–	–	–	20%
S55	+	–	–	–	20%
S82	+	–	–	–	20%
S165	+	–	–	+	40%
S330	–	–	–	+	20%
S550	+	–	–	+	40%
S1000	+	+	+	+	100%
S1600	+	+	+	+	100%

*+ is a positive signal*.

*– is a negative signal*.

Since colorimetric reactions demonstrated to be easily and rapidly analyzed by eye. We next aimed to establish the limits of this detection system. We analyzed the LOD of colorimetric LAMP reactions. For this end, we spiked samples of a nasal swab with known numbers of N-gene synthetic transcripts, and inactivated and lysed these samples. The LOD obtained in these spiked samples was of 66 copies/μL, like the LOD obtained by real-time fluorescence (Figure 2B). We found that an N-gene reaction with 320 copies/μL changed the color of the reaction toward yellow, in contrast with the NTC reaction that remained pink (Figure 2C).

**Figure 3:**
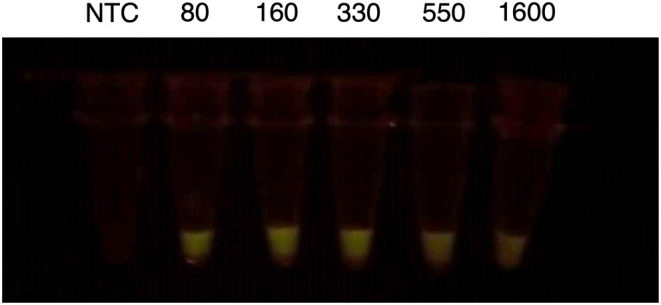
Visual results of dCas12-mediated detection. The results show a representative image of the fluorescence signals in dCas12a reactions. As observed, positive signals glow with a green color under blue-light illumination. However, negative signals have a very faint glow.

Finally, to decrease the limit of detection of the LAMP detection system we proved another fluorescence detection system based on LbCas12a reactions. LbCas12a is a bacterial endonuclease that recognizes and hydrolyzes a target DNA sequence complementary to the sequence of a guide RNA (gRNA). Interestingly, once LbCas12a recognizes and hydrolyzes its target sequence, it activates an endonuclease domain that degrades any DNA molecule at hand (collateral nuclease activity). To use this property of LbCas12a we designed two gRNAs targeting N-gene and POP7, and we amplified the signal by the addition of a quenched DNA-probe, that fluoresce once degraded by activated LbCas12a. For LbCas12a reactions we added 5 μL of LAMP reactions previously incubated for 30 min. Interestingly, the recording of real-time fluorescence of LbCas12a showed that this detection system improves the LOD of the simple LAMP reaction measured by real-time PCR to 16 copies/μL (Table 5). It also improved the visual detection of the LAMP reaction using a blue-light transilluminator (Figure 3).

**Table 5:** Limit of detection of LbCas12a reaction by final fluorescence.

**Positivity of signal**																
**Sample**	**N1**	**N2**	**N3**	**N4**	**N5**	**N6**	**N7**	**N8**	**N9**	**N10**	**N11**	**N12**	**N13**	**N14**	**N15**	**% detection**
NTC	–	–	–	–	–	–	–	–	–	–	–	–	–	–	–	0%
S40	+	+	+	+	–	–	–	–	–	–	–	–	–	–	–	26%
S80	+	+	+	+	+	+	+	+	+	+	+	+	+	+	+	100%
S160	+	+	+	+	+	+	+	+	+	+	+	+	+	+	+	100%
S330	+	+	+	+	+	+	+	+	+	+	+	+	+	+	+	100%
S550	+	+	+	+	+	+	+	+	+	+	+	+	+	+	+	100%
S1600	+	+	+	+	+	+	+	+	+	+	+	+	+	+	+	100%

*+ is a positive signal*.

*– is a negative signal*.

**Figure 4:**
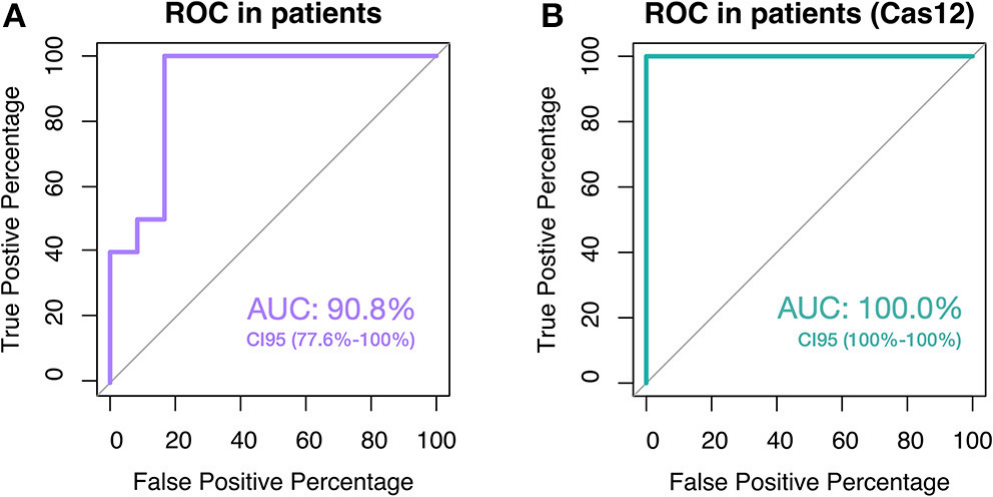
ROC curves and AUC values in the test performed with patient samples. **(A)** The ROC curve and AUC value were calculated with the Ct values of the fluorescent LAMP reactions using patient samples previously validated by Real-time PCR method. **(B)** The ROC curve and AUC value consider the qualitative results of Cas12a enhancement used in validated patient samples.

At last, we proved the efficiency of our SARS-CoV-2 detection system on patient samples previously analyzed by RT-qPCR. As shown in Table 6, LAMP reactions showed good proficiency in detecting SARS-CoV-2 viral transcripts on patient samples. However, using qPCR as a standard, we observed the fluorescent LAMP system failed to detect two of twelve negative samples. Furthermore, the fluorescent LAMP system showed an accuracy of ten of ten in positive samples with which we obtain an AUC value of the ROC curve of 90.8%, CI 95 = 77.6–100% (Figure 4).

**Table 6:** LAMP efficiency in patient samples.

**Sample**	**Origin**	**qPCR result**	**LAMP result**
1	Extraction	Negative	Negative
2	Extraction	Negative	Negative
3	Nasal swab	Negative	Negative
4	Nasal swab	Negative	Negative
5	Nasal swab	Negative	Negative
6	Nasal swab	Negative	Negative
7	Nasal swab	Negative	Negative
8	Nasal swab	Negative	Positive
9	Nasal swab	Negative	Positive
10	Saliva	Negative	Negative
11	Saliva	Negative	Negative
12	Saliva	Negative	Negative
13	Extraction	Positive	Positive
14	Extraction	Positive	Positive
15	Nasal swab	Positive	Positive
16	Nasal swab	Positive	Positive
17	Nasal swab	Positive	Positive
18	Nasal swab	Positive	Positive
19	Nasal swab	Positive	Positive
20	Nasal swab	Positive	Positive
21	Nasal swab	Positive	Positive
22	Nasal swab	Positive	Positive

*Nasal swab: Sample collected from nasal mucosa. Saliva: Collected from the saliva accumulation after 30 s of movements like chewing gum. Extraction: Samples extracted with a commercial kit before the process*.

The results obtained to this point suggest that LAMP reactions are a robust method for SARS-CoV-2 detection. Since amplification can be detected by several methods, being the colorimetric method the most versatile for field applications, and the fluorescent method in addition to the LbCas12a system the more sensible and easier to interpret if the hospital facility counts with a real-time thermocycler (Table 7). This system improves the ROC curve reaching an AUC value of 100.0%, CI 95 = 100–100% using patient samples previously validated with the standard method, qPCR (Figure 4). In addition, LAMP reactions are suitable for the direct use of samples from rapid lysis methods as the ones described here.

**Table 7:** Cas12a efficiency in patient samples.

**Sample**	**Origin**	**qPCR result**	**Cas12a result**
1	Nasal swab	Negative	Negative
2	Nasal swab	Negative	Negative
3	Nasal swab	Negative	Negative
4	Nasal swab	Negative	Negative
5	Nasal swab	Negative	Negative
6	Nasal swab	Negative	Negative
7	Nasal swab	Positive	Positive
8	Nasal swab	Positive	Positive
9	Nasal swab	Positive	Positive
10	Nasal swab	Positive	Positive
11	Nasal swab	Positive	Positive
12	Nasal swab	Positive	Positive

*Nasal swab: Sample collected from nasal mucosa*.

**Figure 5:**
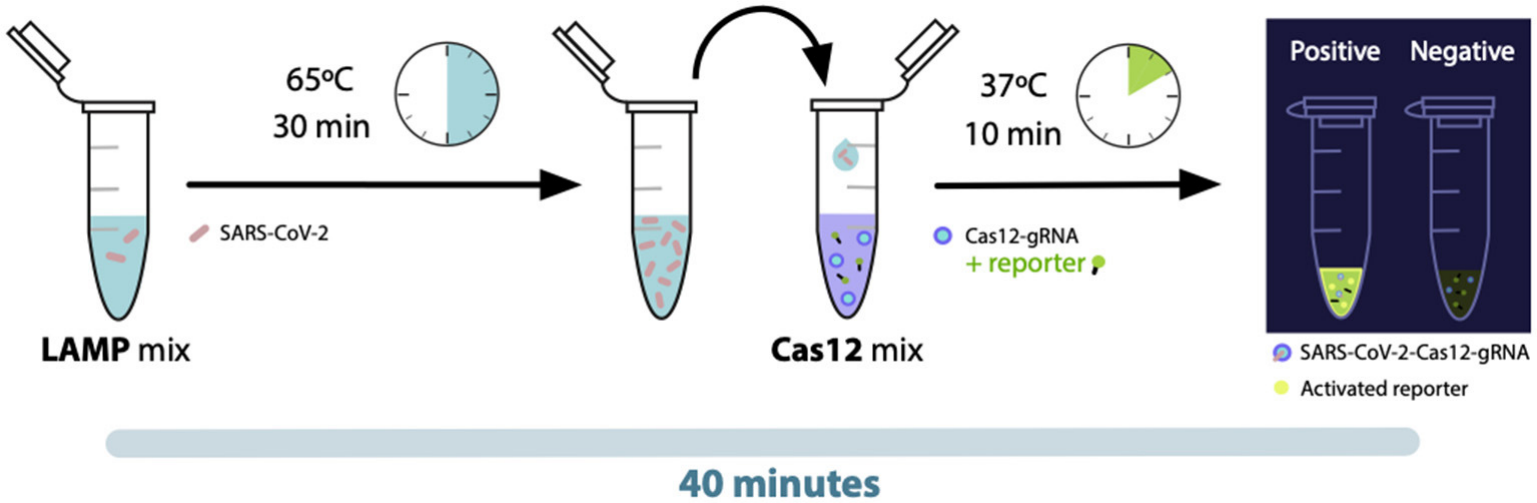
LAMP-Cas12 diagnostic method for SARS-CoV-2 detection. Schema shows the steps to perform the virus detection. From a previous inactivated clinical sample, we add the LAMP mix containing specific primers that target the virus N-gene and amplify it, if is present on the sample. At the same time, the LbCas12a enzyme is incubated with a N-gene specific gRNA, after 30 min the reporter containig a fluorophore is added. When the LbCas12a-gRNA complex finds its target, it has a non-specific cleavage of the reporter that releases the fluorophore and emits fluorescence.

Our LAMP/Cas12 detection protocol is a simple and versatile method that has the advantage of obtaining a result in only 65 min, since the reception of the sample. The procedure consists in a sample treatment with lysis buffer at two temperatures that can be achieved by almost all thermoblocks (20 min at 42 C, and 5 min at 65 C). Followed by the amplification of SARS-CoV-2 N gene by LAMP reactions, which is accomplished in 30 min, and finally the detection of specific amplicons using a Cas12a/crRNA system that can be achieved in 10 min. The readout of the result can be done using a real-time thermocycler, or a blue-light lamp in obscure conditions (Figure 5).

## Discussion

The COVID-19 outbreak has become one of the most important public health problems at the global level. In the absence of an approved vaccine, early and reliable detection of the virus in the entire population is our best strategy to contain COVID-19 lethality and hospitable saturation. In the present work, we developed a new SARS-CoV-2 detection test which allows rapid detection of viral N gene transcript after a simple, fast and cheap RNA isolation procedure. Furthermore, LAMP technology enables a versatile diagnostic test, which can be done using any water bath able to warm at 65°C, and can be detected, in the best of cases with a real-time thermocycler, but also with the naked eye if the equipment is absent.

In this work, we tested several detection methods to develop a more versatile and sensitive diagnostic test for the COVID-19 virus. Based on fluorescence and colorimetric readouts we found that the colorimetric system is the more versatile and easily field-deployable strategy but has the drawbacks that in low-copy number samples the color change is difficult to perceive clearly, and its sensitiveness to bad buffered samples. However, real-time fluorescence detection gave the more easily interpretable results but requires the use of a real-time PCR thermocycler, which is almost impossible to obtain outside of a few facilities. Also, to increase the sensitivity of our diagnostic test, we evaluated the integration of LbCas12a to amplify the signal generated by the LAMP reaction. However, the results obtained showed that fluorescence increase due to LbCas12a activity (due to LAMP amplicons) has a linear behavior, which amplifies more the LAMP signals. Hence, the addition of LbCas12a improves the LOD obtained from LAMP reactions alone.

In comparison with other diagnostic technologies assessed by other groups, our SARS-CoV-2 detection system outperforms almost all currently available diagnostic systems in terms of speed, since the inactivation protocol used decreased RNA-extraction time to 25 min. The developed LAMP system requires just 40 min to obtain a result and if we implement the LbCas12a system we add 10 more minutes to the protocol, much less time compared with the 4 h used in the CDC/WHO protocols (8, 9) and is similar to other LAMP protocols (30–45 min) (6, 10), or Crispr based protocols such as DNA Endonuclease Targeted CRISPR *Trans* Reporter (DETECTR) (30 min) or Specific High Sensitivity Enzymatic Reporter Unlocking (SHERLOCK) (58 min), these methods use a similar RNA extraction protocol (11). However, since the other diagnostic technologies use a time-expensive RNA-extraction protocol, our rapid-inactivation protocol to extract nucleic acids gives us a total time of 65–75 min to obtain a result, starting from sample reception. Also, the RNA extraction protocol required by all methods could represent a risk of exposure and contamination. In this case, we reduce sample processing by using the sample directly.

It is important to note, that the colorimetric LAMP system has several advantages concerning other systems in terms of its applicability. The incubation temperatures used in the entire protocol can be achieved using a common water bath, and the final readout of the test can be easily obtained by visual inspection. Also, our experience revealed that colorimetric LAMP master-mixes (with primers) can be stored at 4°C for up to a week. These advantages make colorimetric LAMP tests a promising technology to improve viral diagnosis in the field, easily adaptable to physicians (3) needs. However, since colorimetric LAMP readout is achieved by a pH decrease, these reactions are sensitive to samples without a good buffering system, giving rise to increased false-negative rates.

The SARS-CoV-2 LAMP diagnostic system developed showed a sensibility of 66 copies per μL, which is comparable to the LOD of DETECTR (70–300 copies per μL) (3) or SHERLOCK (10–100 copies per μL) (11). And if we use the LbCas12a system, this LOD improves to 16 copies per μL. In comparison with other LAMP-based diagnostic methods, our system has a similar sensitivity, since Zhang et al., reported a sensitivity of 24 copies/uL (2, 11, 12), and Yu et al. reported a sensitivity of 10 copies/mL (13) which makes these colorimetric tests almost as sensitive as qPCR. However, the RT-qPCR protocol (CDC/WHO) is still the most sensitive diagnostic method, the limit of detection is about 1–3 copies per μL (4, 8). Furthermore, the LOD value achieved in our diagnostic system showed a good proficiency for the detection of the SARS-CoV-2 virus in patient samples and showed similar results than the qRT-PCR system in the same set. Also, even when the LOD is higher than the conventional method, it is enough to detect infection from clinical samples in which the copy number on average is 676 copies per μL (12). The present method has the advantage of combining two assays without RNA extraction, which, in our hands gives a more robust result that either one separately, as it is more sensitive and specific, while conserving specific technical benefits useful for deploying in the daily lab. For example, the recently published Pang et al. method (14) uses the same tube for the reaction but requires previous RNA extraction and a higher level of manipulation, since each tube has to hold the Cas12 mix in the lid, which is prone to errors when working with multiple samples. The recently published assay by Lalli et al. (15) does not need previous RNA isolation, but starts with saliva, which, in our hands at least, have a reproducibility and specificity issues, as we have found that the additional use of Cas12a decrease false positives. In addition, their method requires the addition of proteinase K or guanidine isothiocyanate, adding to the cost of the assay. Finally, in terms of specificity the SARS-Cov-2 LAMP detection system we present showed no amplification in samples containing human RNA, and the genomes of other viruses causing respiratory diseases. A comparison between assays is provided in Table 8.

**Table 8:** Recent SARS-CoV2 detection method comparison.

	**Our work**	**Wang et al. (16)**	**Pang et al. (14)**	**Lalli et al. (15)**
Methods	RT-LAMP/Cas12a	RT-LAMP/Cas12a	RT-LAMP/Cas12a	RT-LAMP
Time	40 min	45 min	40 min	25 min
LOD	330 cp/rxn (66 cp/μL)	5 cp/μL	150 cp/rxn (30 cp/μL)	59 cp/rxn
Clinical samples	34	50	100 swabs	30 saliva
RNA extraction	No	Yes	Yes	No
Visualization	Colorimetric and fluorescence	Blue light	UV light and cellphone	Colorimetric and fluorescence
Target	N gene	S-gene	N and E gene	N gene and Orflab
Brief description	We use HUDSON (25 min) Both reactions at the same time in separate tubes and mixing the last 10 min.	Both reactions in the same tube and the same time. The LAMP mix in the bottom and the Cas12 mix on the lid for the first incubation, then they mix and do the second incubation.	Similar to Wang without the oil and with different times and volumes	There are two temperature incubation of the samples (65°/15 min and 95°/5 min) followed by an incubation of 25 min.
Volumes	24 μL RT-LAMP + 20 μL Cas12 + 5 μL sample	40 μL RT-LAMP + 20 μL Cas12 + 1 μL sample	25 μL RT-LAMP + 10 μL Cas12 + 2-10 μL sample	20 μL RT-LAMP + 3 μL sample

In conclusion, these results imply that SARS-CoV-2 LAMP detection system showed an average sensitivity in terms of LOD value, but with a proficiency enough to detect the virus in patient samples, and with a high specificity that does not cross with human transcriptomes, or other viral genomes., SARS-CoV-2 LAMP detection system offers a rapid, sensitive and specific option with minimal sample processing or equipment requirements for the detection of SARS-CoV-2.

## Data Availability Statement

The original contributions presented in the study are included in the article/Supplementary Material, further inquiries can be directed to the corresponding author/s.

## Ethics Statement

The studies involving human participants were reviewed and approved by Instituto Nacional de Medicina Genómica Ethics Committee. The patients/participants provided their written informed consent to participate in this study.

## Author Contributions

AG-V, BR-Z, EM-G, AM-M, and HO performed all the experiments and including samples' analysis. VM, IL, and JM-Z coordinated the study. AG-V, BR-Z, VM, and JM-Z wrote the article. AG-V, BR-Z, EM-G, VM, and JM-Z analyzed the data. All authors contributed to the article and approved the submitted version.

## Conflict of Interest

The authors declare that the research was conducted in the absence of any commercial or financial relationships that could be construed as a potential conflict of interest.
